# Beyond BMI: L4–L5 visceral-to-subcutaneous fat ratio on CT predicts impaired wound healing after posterior lumbar fusion—A retrospective study

**DOI:** 10.3389/fsurg.2026.1757071

**Published:** 2026-06-15

**Authors:** Jinwang Liu, Xiaoping Xu, Shaoxing Li, Hua Yu

**Affiliations:** 1 Department of Orthopedics, The First Affiliated Hospital of Chengdu Medical College, Sichuan, China; 2 Department of Gynaecology and Obstetrics, The First Affiliated Hospital of Chengdu Medical College, Sichuan, China

**Keywords:** posterior lumbar fusion, subcutaneous fat area, surgical site complications, VFA/SFA ratio, visceral fat area

## Abstract

**Background:**

Obesity is a recognized risk factor for postoperative complications after spine surgery, but body mass index (BMI) does not capture regional fat distribution. The visceral-to-subcutaneous fat area (VFA/SFA) ratio measured on CT reflects an unfavorable fat distribution pattern, yet its role in wound healing after posterior lumbar fusion (PLF) is unclear.

**Methods:**

We retrospectively analyzed 526 adults who underwent instrumented PLF for degenerative lumbar pathology and had preoperative non-contrast abdominal CT including the L4–L5 level. On a single axial slice at L4–L5, visceral fat area (VFA) and subcutaneous fat area (SFA) were segmented using a semi-automated protocol and the VFA/SFA ratio was calculated and grouped into quartiles. The primary outcome was impaired wound healing within 30 days, defined as persistent erythema or swelling, fat liquefaction, prolonged serous/serosanguinous exudation, partial wound edge necrosis or superficial dehiscence, or the need for negative-pressure therapy, delayed suture removal, additional antibiotics, or surgical debridement. Group comparisons used t tests and *χ*^2^ tests. Univariable and multivariable logistic regression were performed, and discrimination of BMI, VFA, SFA, VFA/SFA, and clinical prediction models was assessed using receiver operating characteristic curves.

**Results:**

Impaired wound healing occurred in 59 patients (11.2%). Compared with patients with good healing, those with impaired wound healing had higher BMI (25.4 ± 4.0 vs. 23.1 ± 3.8 kg/m^2^, *p* < 0.001) and higher VFA/SFA ratio (1.063 ± 0.246 vs 0.895 ± 0.259, *p* < 0.001), whereas SFA did not differ. Each 0.1 increase in VFA/SFA ratio was associated with an adjusted odds ratio of 1.32 (95% CI 1.16–1.50; *p* < 0.001) for impaired wound healing, independent of age, sex, BMI, diabetes, ASA class, albumin, multilevel fusion, operative time, and blood loss. The AUC was 0.66 for BMI and 0.70 for VFA/SFA; adding VFA/SFA to a clinical model increased the AUC from 0.70 to 0.77.

**Conclusion:**

The preoperative VFA/SFA ratio at L4–L5 is an independent predictor of impaired wound healing after PLF and provides incremental discriminative value beyond BMI and conventional clinical risk factors.

## Introduction

Posterior lumbar fusion (PLF) is widely performed to treat degenerative lumbar conditions, including spinal stenosis, spondylolisthesis, and segmental instability (1). Clinical outcomes are generally favorable, but postoperative complications remain a major concern for both patients and surgeons. Among these, wound-related complications such as impaired wound healing, fat liquefaction, and surgical site infection (SSI) are particularly troublesome because they may require prolonged wound care, reoperation, or even implant removal, and they substantially increase healthcare costs (2).

Obesity is a well-recognized risk factor for perioperative complications in spine surgery. However, body mass index (BMI) is an imperfect surrogate for adiposity (3–5). BMI does not differentiate between lean and fat mass and does not reflect regional patterns of fat distribution (6). There is increasing evidence that regional adiposity metrics, such as posterior subcutaneous fat thickness, paraspinal muscle quality, and abdominal visceral fat, may provide more clinically meaningful risk information than BMI alone (7, 8).

Visceral adipose tissue is metabolically active and secretes proinflammatory cytokines and adipokines that contribute to systemic low-grade inflammation, insulin resistance, and endothelial dysfunction (9, 10). In contrast, subcutaneous adipose tissue is relatively metabolically benign and may even exert protective effects in some settings (11). Therefore, the balance between visceral and subcutaneous fat depots, rather than the absolute amount of visceral fat, may be critical for tissue repair and wound healing (12).

Several studies in abdominal, orthopedic, and spine surgery have shown that increased VFA and greater posterior subcutaneous fat are associated with higher rates of wound complications and SSI (13, 14). Nevertheless, the role of the visceral-to-subcutaneous fat area ratio (VFA/SFA) as a composite index of regional fat distribution has not been fully explored in the context of spine surgery (15). The VFA/SFA ratio has been used in cardiometabolic research as an indicator of an “unfavorable” fat distribution pattern and has been associated with an increased risk of diabetes, cardiovascular disease, and mortality (16, 17).

Accurate assessment of VFA and SFA relies on cross-sectional imaging. Single-slice CT measurements at the L4–L5 intervertebral level are widely used to approximate total abdominal visceral adipose tissue volume and correlate well with cardiometabolic risk factors (18). Lumbar spine imaging at or near L4–L5 is routinely obtained in candidates for PLF, which provides a practical opportunity to quantify VFA and SFA without additional imaging (19).

The primary objective of this study was to determine whether the preoperative VFA/SFA ratio at L4–L5 is associated with impaired wound healing after PLF. We hypothesized that a higher VFA/SFA ratio would be independently associated with impaired wound healing and that it would add predictive value beyond BMI and conventional clinical risk factors.

## Methods

### Study design

This was a retrospective cohort study performed at a tertiary spine referral center. The institutional review board approved the study protocol(IRB No. 2025CYFYIRB-BA-161). Informed consent was obtained from all participants in the study. All protocols were conducted in accordance with the research principles set forth in the Declaration of Helsinki.

### Patient selection

We screened all adult patients (≥ 18 years) who underwent instrumented PLF between May 2017 and March 2025. Inclusion criteria were: 1) PLF for degenerative lumbar spine pathology (spinal stenosis, spondylolisthesis, disc degeneration, or degenerative deformity). 2) Availability of preoperative abdominal CT including the L4–L5 intervertebral level within 3 months of surgery. 3) Minimum follow-up of 30 days after surgery.

Exclusion criteria were: 1) Surgery for acute trauma, tumor, or active spinal infection. 2) Revision PLF procedures. 3) Combined anterior or lateral approaches during the same index surgery. 4) Known systemic inflammatory or autoimmune disease that might independently affect wound healing. 5) Incomplete clinical, laboratory, or imaging data.

After applying these criteria, a total of 526 patients were included in the final cohort.

### Surgical technique and perioperative management

All procedures were performed under general anesthesia by fellowship-trained spine surgeons following standard posterior midline approaches. After midline incision, subperiosteal dissection exposed the posterior elements. Decompression, facetectomy, and interbody fusion were performed as indicated. Pedicle screws and rods were placed under fluoroscopic guidance. Posterolateral fusion with autograft and/or bone graft substitutes was performed in all patients combined with interbody cages.

The wound was irrigated with saline and hemostasis was achieved before layered closure of fascia, subcutaneous tissue, and skin. Closed-suction drains were used according to the surgeon's preference and typically removed when drainage was <50 mL over 24 h, usually within 48–72 h. Perioperative antibiotic prophylaxis followed institutional protocols, typically a first-generation cephalosporin administered within 30 min before incision and continued for 24 h postoperatively.

Standard perioperative measures to reduce SSI and wound complications, including optimization of glucose control in patients with diabetes, maintenance of normothermia, and smoking cessation counseling, were implemented as part of routine clinical care.

### Definition of impaired wound healing

The primary outcome was impaired wound healing within 30 days postoperatively. Impaired wound healing was defined as clinically meaningful wound problems that prompted unplanned wound-related management, including any of the following: 1) Persistent wound erythema and swelling beyond the expected postoperative course requiring unplanned clinical action (e.g., aspiration/puncture, initiation/escalation of antibiotics, or prolonged non-routine dressing changes). 2) Fat liquefaction or subcutaneous induration/fluctuance requiring aspiration/drainage or additional wound care. 3) Serous or serosanguinous exudation requiring prolonged dressing changes. 4) Partial wound edge necrosis or superficial dehiscence. 5) Need for negative pressure wound therapy, delayed suture removal, additional antibiotics, or surgical debridement. This intervention-based definition aligns with prior descriptions of postoperative wound healing problems after spine surgery (20).

Patients without any of the above findings, whose wounds healed within the expected timeframe without additional interventions, were classified as having good wound healing.

### Clinical and laboratory variables

Baseline demographic and clinical variables included age, sex, BMI, smoking status, and comorbidities (including diabetes mellitus). ASA physical status classification was recorded from anesthesia records and dichotomized as ASA I–II versus ASA III–IV.

Preoperative serum albumin levels were measured within 7 days of surgery. Hypoalbuminemia was defined as albumin < 3.5 g/dL. Operative variables included primary diagnosis, number of fused levels (single vs. ≥ 2 levels), operative time (minutes from skin incision to closure), and intraoperative estimated blood loss (mL).

### Imaging acquisition and body composition analysis

Preoperative imaging consisted of clinically indicated non-contrast CT scans that included the L4–L5 intervertebral level. Axial images at the L4–L5 disc level were identified using sagittal scout images and vertebral body morphology, and a single 5-mm slice at this level was selected for analysis in each patient. All examinations were reconstructed with a standard soft-tissue kernel at 120 kVp. No additional CT examinations were performed solely for the purpose of VFA/SFA measurement. In our clinical workflow, the routinely reported spine series is often reconstructed/cropped to a smaller spine-focused field; therefore, VFA/SFA was quantified from the original uncropped DICOM source data/large-FOV soft-tissue reconstruction at L4–L5, which includes the full abdominal wall circumference required for segmentation.

Body composition analysis was performed on the L4–L5 axial slice using SliceOmatic (version 5.0, Canada). Adipose tissue was identified by applying a predefined Hounsfield unit (HU) threshold from −190 to −30 HU, a range that has been widely used and validated for CT-based quantification of abdominal fat (21, 22). Voxels within this attenuation range were classified as fat, whereas voxels outside this range (e.g., skin, skeletal muscle, viscera, and bone, which typically exhibit attenuation values above 30 HU) were excluded.

**Figure 1:**
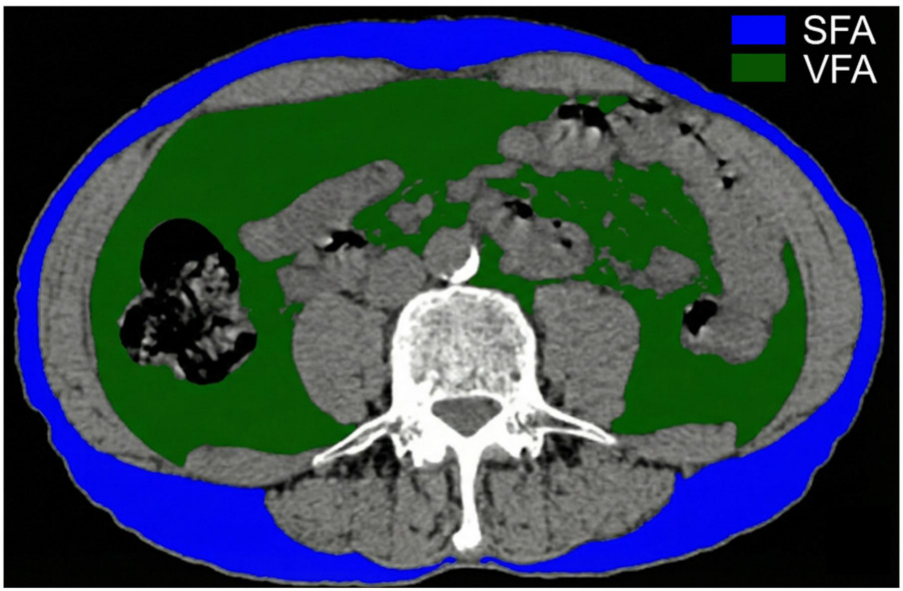
Representative axial non-contrast CT image at the L4–L5 intervertebral level illustrating segmentation of abdominal adipose tissue. The blue overlay denotes subcutaneous fat area (SFA), the green overlay denotes visceral fat area (VFA).

For each scan, two adipose tissue compartments were delineated. Visceral fat area (VFA) was defined as adipose tissue within the abdominal cavity bounded by the inner margin of the abdominal muscular wall and the posterior paravertebral muscles. Subcutaneous fat area (SFA) was defined as adipose tissue between the skin surface and the external border of the abdominal musculature and fascia (Figure 1). Semi-automated segmentation tools were used to generate initial contours, which were then manually refined as needed by the operator.

The areas of VFA and SFA were calculated in square centimeters (cm^2^), and the VFA/SFA ratio was derived for each patient. The distribution of the VFA/SFA ratio in the entire cohort was used to define quartiles (Q1–Q4), with Q1 representing the lowest and Q4 the highest ratio.

To assess measurement reproducibility, two experienced radiologists independently measured VFA and SFA on a random sample of 50 CT scans, blinded to clinical outcomes. The intraclass correlation coefficient (ICC) for interobserver agreement was 0.893.

### Statistical analysis

All analyses were performed using SPSS 23.0 (SPSS, IBM Analytics, New York, USA) and the figures were created by GraphPad Prism version 9.3.0 (GraphPad Software, San Diego, California USA). Patients were categorized into two groups based on wound outcome: impaired wound healing vs good wound healing. Continuous variables were summarized as mean ± standard deviation (SD) and compared using Student's t-test. Categorical variables were summarized as counts and percentages and compared using *χ*^2^ tests or Fisher's exact test as appropriate.

Univariable logistic regression analyses were performed to evaluate the association between impaired wound healing and the related factors. Variables with *p* < 0.10 on univariable analysis, together with clinically relevant factors, were considered for inclusion in a multivariable logistic regression model. The primary predictor of interest was the VFA/SFA ratio. Adjusted odds ratios (ORs) and 95% confidence intervals (CIs) were reported.

ROC curves were used to assess the discriminative ability. A base clinical model was constructed including age, sex, BMI, diabetes mellitus, ASA class, multilevel fusion, operative time, and hypoalbuminemia. A second model included the same variables plus the VFA/SFA ratio. AUCs were calculated for both models to evaluate the incremental predictive value of VFA/SFA ratio. The optimal cutoff value for VFA/SFA ratio was determined using the Youden index. In addition, given known sex-related differences in body fat distribution, we performed sex-stratified descriptive analyses for VFA, SFA, and VFA/SFA ratio, and tested a sex-by-VFA/SFA interaction term in the multivariable model to assess possible effect modification. All statistical tests were two-sided, and a p value < 0.05 was considered statistically significant.

## Results

### Patient characteristics

A total of 526 patients met the inclusion criteria. Among them, 59 (11.2%) experienced impaired wound healing within 30 days of PLF, while 467 had good wound healing.

Baseline characteristics stratified by wound outcome are presented in Table 1. Age and sex distribution were similar between groups (mean age 63.3 ± 10.0 vs. 63.7 ± 11.3 years, *p* = 0.79; female sex 55.9% vs 53.7%, *p* = 0.86). Patients with impaired wound healing had significantly higher BMI (25.4 ± 4.0 vs. 23.1 ± 3.8 kg/m^2^, *p* < 0.001). The prevalence of diabetes mellitus was higher in the impaired group (47.5% vs 34.9%), although this difference did not reach statistical significance (*p* = 0.08).

**Table 1:** Baseline characteristics of the study cohort.

Variable	Impaired wound healing (*n* = 59)	Good healing (*n* = 467)	*p* value
Age (years), mean ± SD	63.3 ± 10.0	63.7 ± 11.3	0.79
Female sex, n (%)	33 (55.9)	251 (53.7)	0.86
BMI (kg/m^2^), mean ± SD	25.4 ± 4.0	23.1 ± 3.8	<0.001
Diabetes mellitus, n (%)	28 (47.5)	163 (34.9)	0.08
Current smoker, n (%)	20 (33.9)	119 (25.5)	0.22
ASA class III–IV, n (%)	15 (25.4)	106 (22.7)	0.76
Albumin <3.5 g/dL, n (%)	14 (23.7)	58 (12.4)	0.03
Multilevel fusion ≥2 levels, n (%)	28 (47.5)	204 (43.7)	0.68
Operative time (min), mean ± SD	212 ± 79	205 ± 68	0.47
Estimated blood loss (mL), mean ± SD	482 ± 300	442 ± 243	0.25
Drain use	53 (89.8)	425 (91)	0.81

The frequency of current smoking was 33.9% in the impaired group and 25.5% in the good healing group (*p* = 0.22). Proportions of patients with ASA class III–IV were similar (25.4% vs 22.7%, *p* = 0.76). Hypoalbuminemia was more frequent among patients with impaired wound healing (23.7% vs 12.4%, *p* = 0.03). The proportion of patients undergoing multilevel fusion (≥ 2 levels) was slightly higher in the impaired group (47.5% vs. 43.7%), but this was not statistically significant (*p* = 0.68). Operative time estimated blood loss and drain use did not differ significantly between groups.

### Body composition at L4–L5

Body composition parameters at L4–L5 are summarized in Table 2. In the overall cohort, mean VFA was 134.7 ± 73.2 cm^2^ and mean SFA was 147.4 ± 66.6 cm^2^. The mean VFA/SFA ratio was 0.914 ± 0.263.

**Table 2:** Body composition parameters at L4–L5 by wound healing status.

Variable	Impaired wound healing (*n* = 59)	Good healing (*n* = 467)	*p* value
VFA at L4–L5 (cm^2^)	149.3 ± 76.6	132.8 ± 72.6	0.102
SFA at L4–L5 (cm^2^)	140.5 ± 59.8	148.3 ± 67.4	0.398
VFA/SFA ratio	1.063 ± 0.246	0.895 ± 0.259	<0.001
Incidence of impaired wound healing by VFA/SFA quartile			
Q1 (lowest)	5/132 (3.8%)	127/132 (96.2%)	—
Q2	10/131 (7.6%)	121/131 (92.4%)	—
Q3	16/131 (12.2%)	115/131 (87.8%)	—
Q4 (highest)	28/132 (21.2%)	104/132 (78.8%)	—

Patients with impaired wound healing had numerically higher VFA than those with good healing, although the difference did not reach statistical significance (149.3 ± 76.6 vs. 132.8 ± 72.6 cm^2^, *p* = 0.102). In contrast, SFA did not differ between the two groups (140.5 ± 59.8 vs. 148.3 ± 67.4 cm^2^, *p* = 0.398). The VFA/SFA ratio was markedly higher in patients with impaired wound healing (1.063 ± 0.246 vs. 0.895 ± 0.259, *p* < 0.001).

**Figure 2:**
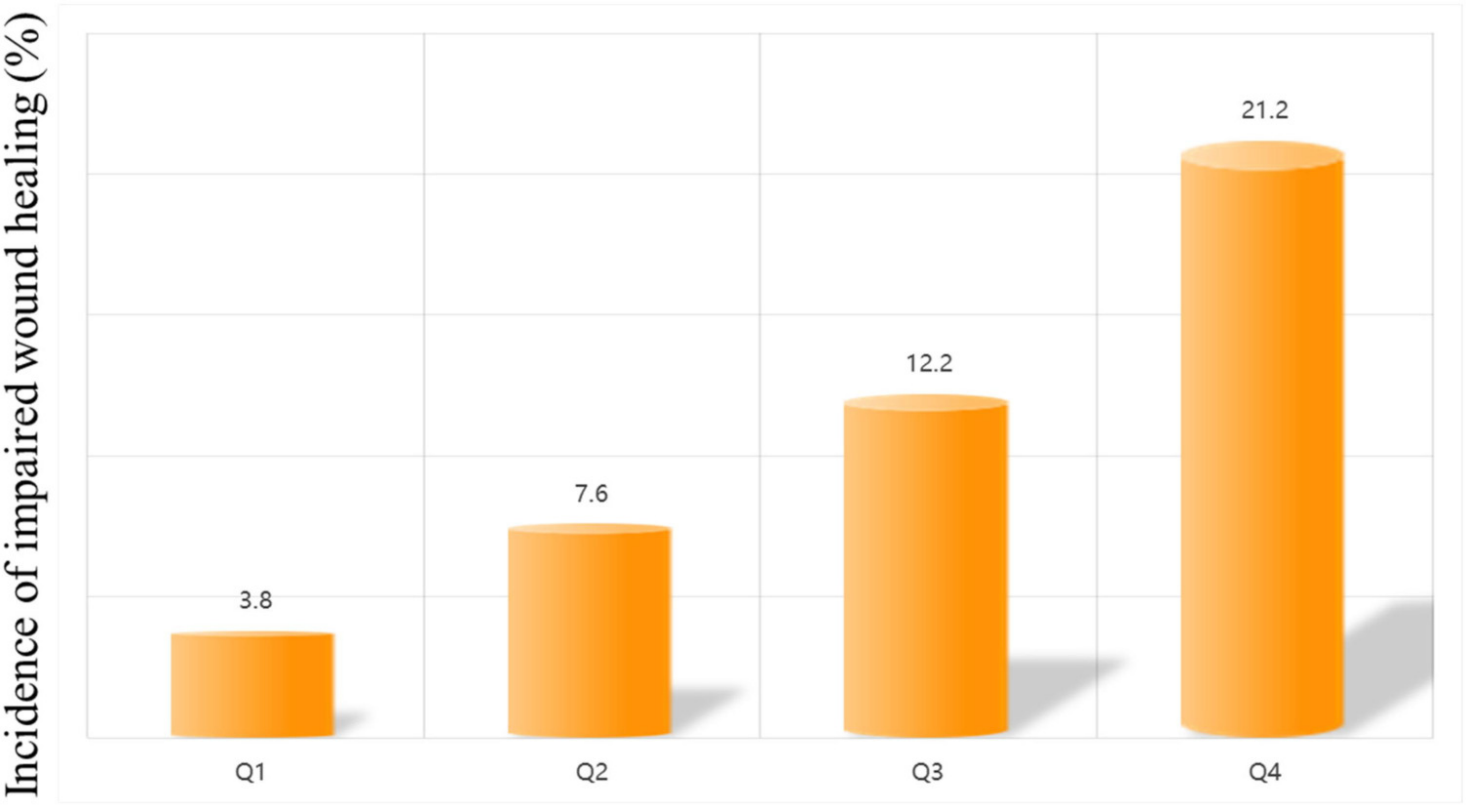
Incidence of impaired wound healing according to quartiles of the VFA/SFA ratio at L4–L5.

When patients were stratified by quartiles of VFA/SFA ratio, the incidence of impaired wound healing increased in a graded fashion: 3.8% in Q1 (lowest ratio), 7.6% in Q2, 12.2% in Q3, and 21.2% in Q4 (highest ratio) (Figure 2).

Because visceral and subcutaneous fat distributions may differ by sex, we additionally performed sex-stratified body-composition analyses (Supplementary Table S1). Female patients had a mean VFA of 129.1 ± 66.6 cm^2^, a mean SFA of 142.6 ± 64.9 cm^2^, and a mean VFA/SFA ratio of 0.913 ± 0.260, whereas male patients had corresponding values of 141.6 ± 80.2 cm^2^, 153.4 ± 68.4 cm^2^, and 0.914 ± 0.267. While men tended to have slightly larger absolute VFA and SFA values, these differences were not statistically significant (VFA *p* = 0.052; SFA *p* = 0.064), and the VFA/SFA ratio was nearly identical between sexes (*p* = 0.977). The incidence of impaired wound healing was 14.0% (41/292) in women and 7.7% (18/234) in men. In sex-stratified multivariable analyses, the association between a higher VFA/SFA ratio and impaired wound healing remained directionally consistent in both women (OR 13.52, 95% CI 2.64–69.37; *p* = 0.002) and men (OR 17.55, 95% CI 2.01–153.14; *p* = 0.010), with no evidence of effect modification by sex (p for interaction = 0.886).

Severity-stratified analysis (Supplementary Table S2) showed that, among the 59 patients with impaired wound healing, mean VFA/SFA ratio increased progressively across grades 1–5 (0.902 ± 0.158, 1.001 ± 0.170, 1.054 ± 0.259, 1.169 ± 0.233, and 1.189 ± 0.190, respectively). Spearman analysis demonstrated a moderate positive correlation between wound grade and VFA/SFA ratio (*ρ* = 0.437, *p* < 0.01), and the distribution differed significantly across grades (Kruskal–Wallis *p* = 0.0195) (Supplementary Figure S1). When severity was dichotomized as mild/moderate (grades 1–3) versus severe (grades 4–5), a higher VFA/SFA ratio was associated with greater odds of severe wound breakdown (OR 43.89, 95% CI 3.12–618.19; *p* = 0.005).

### Univariable logistic regression

Results of univariable logistic regression are shown in Table 3. When modeled as a continuous variable, the VFA/SFA ratio was strongly associated with impaired wound healing. For each 0.1 increase in VFA/SFA ratio, the OR for impaired wound healing was 1.27 (95% CI 1.14–1.41; *p* < 0.001).

**Table 3:** Univariable logistic regression analysis for impaired wound healing.

Variable	OR	95% CI	*p* value
VFA/SFA ratio (per 0.1 increase)	1.27	1.14–1.41	<0.001
BMI (per 1 kg/m^2^)	1.17	1.09–1.26	<0.001
Diabetes mellitus (yes vs. no)	1.68	0.98–2.91	0.06
ASA class III–IV (vs I–II)	1.16	0.62–2.17	0.64
Albumin <3.5 g/dL (yes vs. no)	2.19	1.13–4.24	0.02
Multilevel fusion ≥2 levels (vs single level)	1.16	0.68–2.00	0.58
Operative time (per 30 min)	1.04	0.93–1.17	0.47
Estimated blood loss (per 100 mL)	1.06	0.96–1.18	0.25

BMI was also significantly associated with impaired wound healing (OR 1.17 per 1 kg/m^2^, 95% CI 1.09–1.26; *p* < 0.001). Hypoalbuminemia increased the odds of impaired wound healing 2.19-fold (OR 2.19, 95% CI 1.13–4.24; *p* = 0.02). Diabetes mellitus showed a positive association (OR 1.68, 95% CI 0.98–2.91; *p* = 0.061), while ASA class III–IV, multilevel fusion, longer operative time, and greater blood loss showed trends in the expected directions but did not reach statistical significance.

### Multivariable logistic regression

Multivariable logistic regression results are presented in Table 4. After adjustment for age, sex, BMI, diabetes mellitus, ASA class, hypoalbuminemia, multilevel fusion, operative time, and blood loss, the VFA/SFA ratio remained an independent predictor of impaired wound healing. The adjusted OR for impaired wound healing was 1.32 per 0.1 increase in VFA/SFA ratio (95% CI 1.16–1.50; *p* < 0.001).

**Table 4:** Multivariable logistic regression analysis for impaired wound healing.

Variable	Adjusted OR	95% CI	*p* value
VFA/SFA ratio (per 0.1 increase)	1.32	1.16–1.50	<0.001
BMI (per 1 kg/m^2^)	1.17	1.08–1.27	<0.001
Diabetes mellitus (yes vs no)	1.63	0.91–2.93	0.10
Albumin <3.5 g/dL (yes vs no)	1.97	0.96–4.06	0.06
ASA class III–IV (vs. I–II)	1.68	0.85–3.32	0.14
Multilevel fusion ≥2 levels (vs. single level)	1.02	0.57–1.83	0.94
Operative time (per 30 min)	1.06	0.94–1.20	0.35
Estimated blood loss (per 100 mL)	1.06	0.95–1.19	0.30
Age (per year)	1.00	0.97–1.03	0.87
Female sex (vs. male)	0.99	0.55–1.80	0.98

BMI also remained an independent risk factor with an adjusted OR of 1.17 per kg/m^2^ (95% CI 1.08–1.27; *p* < 0.001). Diabetes mellitus, hypoalbuminemia, ASA class, multilevel fusion, operative time, blood loss, age, and sex were not independently associated with impaired wound healing after adjustment.

### ROC analysis and model performance

**Figure 3:**
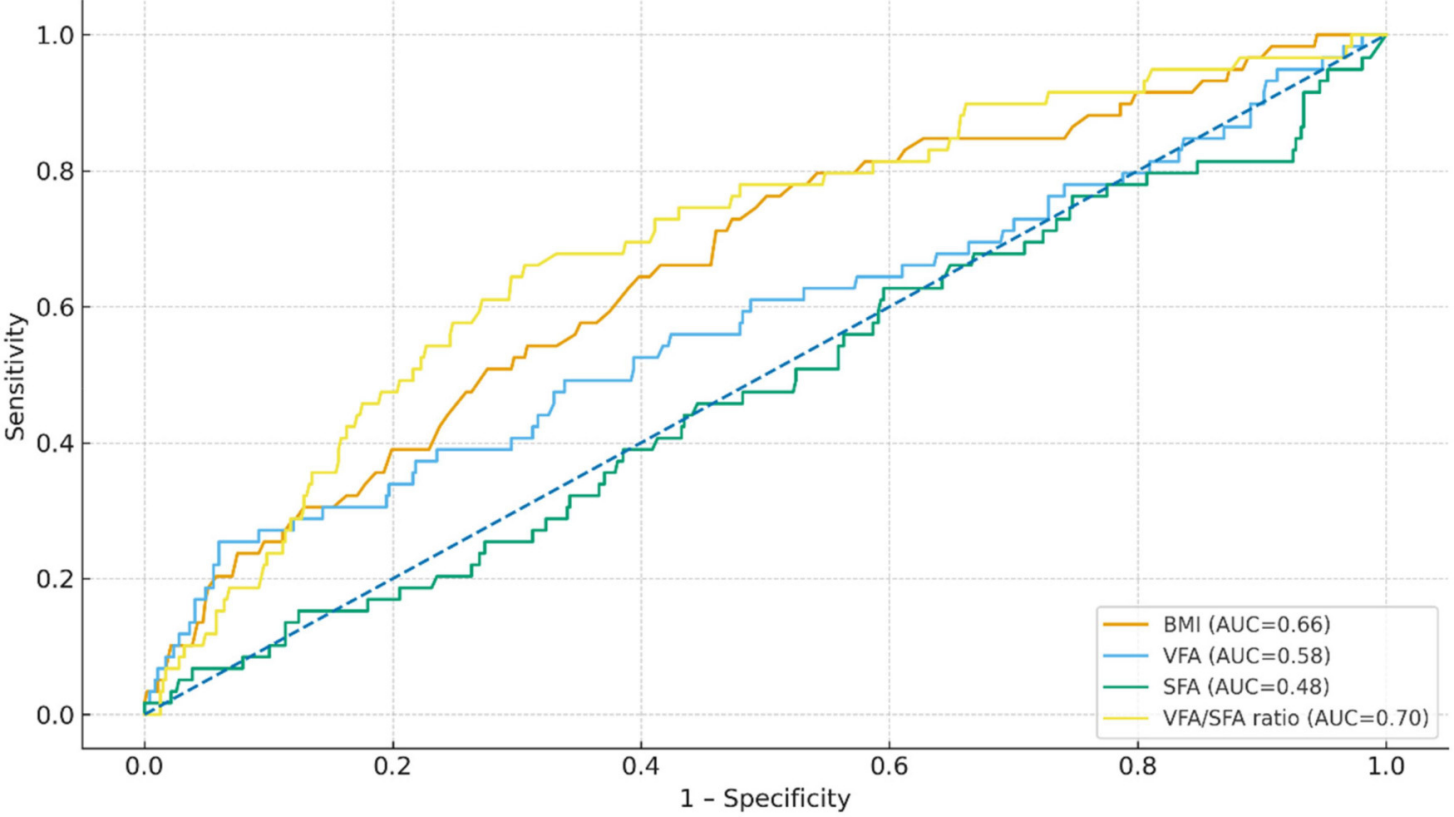
ROC curves for obesity and body composition metrics in predicting impaired wound healing.

ROC curves for BMI, VFA, SFA, and VFA/SFA ratio are shown in Figure 3. The AUCs were: BMI: 0.66, VFA: 0.58, SFA: 0.48 and VFA/SFA ratio: 0.70. Thus, the VFA/SFA ratio provided better discrimination for impaired wound healing than BMI, VFA, or SFA considered individually.

**Figure 4:**
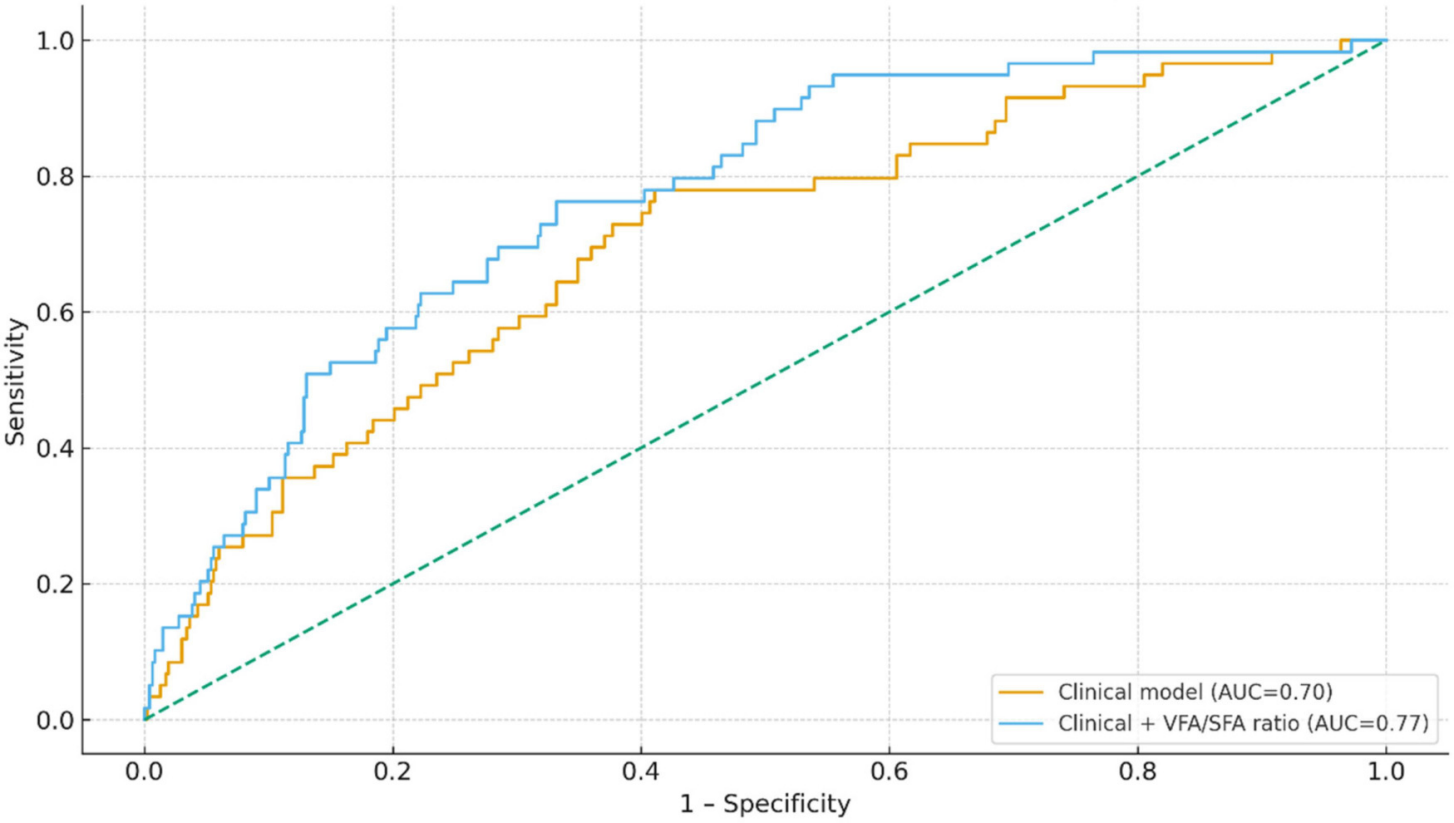
ROC curves for the clinical model with and without the VFA/SFA ratio.

The base clinical model including age, sex, BMI, diabetes mellitus, ASA class, multilevel fusion, operative time, and hypoalbuminemia yielded an AUC of 0.70. When the VFA/SFA ratio was added to this model, the AUC improved to 0.77, indicating a modest but clinically relevant enhancement in discriminative performance (Figure 4).

The optimal cutoff for VFA/SFA ratio determined by the Youden index was approximately 0.76, which corresponded to a sensitivity of 66% and a specificity of 69% for predicting impaired wound healing.

## Discussion

In this retrospective cohort of 526 patients undergoing PLF, we found that the preoperative VFA/SFA ratio measured at L4–L5 was strongly and independently associated with impaired wound healing. Patients in the highest quartile of VFA/SFA ratio had nearly a five-fold higher incidence of impaired wound healing compared with those in the lowest quartile. The association remained significant after adjustment for BMI and other conventional risk factors.

These findings extend previous observations that obesity is linked to wound complications after spine surgery by highlighting the importance of fat distribution, not just overall adiposity. Prior studies have shown that posterior subcutaneous fat thickness and abdominal VFA are associated with a higher risk of SSI after thoracolumbar surgery (23). Our study demonstrates that the balance between visceral and subcutaneous fat depots, as captured by the VFA/SFA ratio, is an even more powerful predictor of wound healing problems after PLF.

The pathophysiological mechanisms underlying this association are likely multifactorial. Visceral adipose tissue is characterized by increased secretion of proinflammatory cytokines, adipokines, and prothrombotic factors, and it is associated with insulin resistance and endothelial dysfunction (24). These systemic alterations can impair tissue perfusion, collagen synthesis, and immune responses at the surgical site, thereby delaying wound healing and promoting fat necrosis (25, 26). In contrast, subcutaneous adipose tissue is relatively metabolically inert and may buffer lipid overflow from the viscera (27). A high VFA/SFA ratio therefore represents a predominance of metabolically adverse visceral fat relative to subcutaneous stores, which may predispose to wound complications (28).

Our data also show that BMI remains an independent risk factor for impaired wound healing, but its discriminative ability is limited compared with VFA/SFA ratio. The AUC for BMI was 0.66, whereas the AUC for VFA/SFA ratio was 0.70. More importantly, adding VFA/SFA ratio to a clinical model containing BMI and other conventional variables improved the AUC from 0.70 to 0.77. This suggests that preoperative risk stratification should consider regional adiposity metrics rather than relying on BMI alone, particularly in patients with a central or visceral obesity phenotype.

Although women had a higher crude incidence of impaired wound healing in our cohort, the VFA/SFA ratio itself was highly similar between sexes, and sex-stratified analyses showed a directionally consistent association between higher VFA/SFA ratio and impaired wound healing, with no evidence of significant effect modification by sex. These findings suggest that the prognostic relevance of the ratio may be relatively robust across sexes, although the subgroup-specific estimates were imprecise because of limited event numbers, particularly in men.

The use of the L4–L5 level for body composition assessment is a practical strength of this study. Single-slice VFA and SFA measurements at L4–L5 or the umbilical level are widely used as surrogates for total abdominal visceral fat and have been validated against volumetric methods. In patients undergoing lumbar spine evaluation, imaging at L4–L5 is routinely available, which means that VFA/SFA ratio can be calculated from existing preoperative scans without additional radiation or cost.

From a clinical standpoint, incorporating VFA/SFA ratio into preoperative assessment has several potential applications. First, it may improve risk communication and patient counseling regarding the likelihood of wound complications and the potential need for prolonged wound care or reoperation. Second, it may help identify patients who warrant closer preoperative assessment and individualized consideration of established optimization measures, although whether interventions targeting visceral adiposity or metabolic status can reduce wound complications remains to be confirmed in prospective studies. Third, in patients with particularly unfavorable VFA/SFA ratios, surgeons may choose to implement more intensive wound management strategies, including meticulous soft tissue handling, layered closure reinforcement, or prophylactic negative-pressure wound therapy.

A severity analysis further suggested that higher VFA/SFA ratio tracked with increasing wound grade, with a moderate positive Spearman correlation and higher odds of severe (grade 4–5) versus mild/moderate (grade 1–3) wound problems. These findings highlight that body fat distribution, rather than overall adiposity alone, plays a critical role in the progression of wound severity, as the VFA/SFA ratio captures the pathological imbalance between metabolically active visceral adipose tissue and relatively inert subcutaneous adipose tissue.

However, several limitations should be acknowledged. First, the retrospective design is inherently subject to residual confounding and potential misclassification bias. Some mild wound problems may have been underreported, particularly if managed in outpatient settings. Second, we assessed body composition at a single point in time; dynamic changes in adiposity and metabolic status before or after surgery were not captured. In addition, this was a single-center study, which may limit generalizability to other populations or surgical practices. External validation in independent cohorts is needed before the VFA/SFA ratio can be widely adopted as a risk stratification tool. In addition, detailed pre-existing dermatologic/skin conditions (e.g., chronic dermatitis/eczema/psoriasis) and structured preoperative weight-management referrals were not uniformly captured as structured variables in this retrospective dataset, precluding reliable adjustment and leaving the possibility of residual confounding. Although we added sex-stratified descriptive and interaction analyses, subgroup-specific estimates remained imprecise because of the limited number of wound-healing events, particularly among men, future prospective studies with standardized data capture, sex-specific body-composition analyses, and longer follow-up are warranted. In addition, formal preoperative weight-management referral and intervention data were not uniformly available in this retrospective cohort, precluding reliable evaluation of whether such optimization influenced wound-healing outcomes. Future prospective studies should determine whether targeted preoperative metabolic and weight optimization in patients with elevated VFA/SFA ratio can reduce postoperative wound complications.

Despite these limitations, the robustness of the association between VFA/SFA ratio and impaired wound healing, together with the improvement in model performance, suggests that this metric has clinical relevance and warrants further investigation.

## Conclusions

In a cohort of 526 patients undergoing posterior lumbar fusion, the preoperative visceral-to-subcutaneous fat area ratio at L4–L5 was strongly and independently associated with impaired wound healing within 30 days of surgery. These results support the integration of regional body fat distribution, particularly the VFA/SFA ratio, into preoperative risk stratification and optimization strategies for patients undergoing posterior lumbar fusion (14, 15, 24).

## Data Availability

The raw data supporting the conclusions of this article will be made available by the authors, without undue reservation.
