# Supplementary material for: Beyond BMI: L4–L5 visceral-to-subcutaneous fat ratio on CT predicts impaired wound healing after posterior lumbar fusion—A retrospective study
Source: Front Surg. 2026 Jun 15;13:1757071. doi: 10.3389/fsurg.2026.1757071 (PMC13310561; doi:10.3389/fsurg.2026.1757071)
Supplement: Supplementary file 1 [file Datasheet1.docx]

**Supplementary Table S1. Sex-stratified body composition measures and interaction analysis for impaired wound healing**

Values are presented as mean ± SD unless otherwise indicated. Subgroup odds ratios were derived from sex-specific multivariable logistic regression models adjusted for age, BMI, diabetes mellitus, ASA class ≥ III, hypoalbuminemia, multilevel fusion, operative time, and blood loss.

| Variable | Female | Male |
| --- | --- | --- |
| n | 292 | 234 |
| Impaired wound healing, n (%) | 41 (14.0%) | 18 (7.7%) |
| VFA (cm²) | 129.1 ± 66.6 | 141.6 ± 80.2 |
| SFA (cm²) | 142.6 ± 64.9 | 153.4 ± 68.4 |
| VFA/SFA ratio | 0.913 ± 0.260 | 0.914 ± 0.267 |
| Adjusted OR for VFA/SFA ratio | 13.52 | 17.55 |
| 95% CI | 2.64–69.37 | 2.01–153.14 |
| p value | 0.002 | 0.010 |
| Sex difference p value (VFA) | 0.057 | |
| Sex difference p value (SFA) | 0.066 | |
| Sex difference p value (VFA/SFA ratio) | 0.977 | |
| p for interaction (sex × VFA/SFA ratio) | 0.886 | |

**Supplementary Table S2. Exploratory wound severity grades and corresponding body composition measures**

Values are presented as mean ± SD. Wound grades 1–5 were used for teaching/demo severity stratification.

| Wound grade | VFA/SFA ratio | VFA (cm²) | SFA (cm²) | BMI (kg/m²) |
| --- | --- | --- | --- | --- |
| 1 Persistent wound erythema and swelling | 0.902 ± 0.158 | 99.2 ± 37.9 | 108.6 ± 42.6 | 25.06 ± 3.34 |
| 2 Fat liquefaction or subcutaneous induration/fluctuance | 1.001 ± 0.170 | 130.7 ± 56.5 | 133.2 ± 43.3 | 25.75 ± 2.94 |
| 3 Serous or serosanguinous exudation | 1.054 ± 0.259 | 151.2 ± 78.0 | 143.9 ± 50.2 | 27.67 ± 4.00 |
| 4 Partial wound edge necrosis or superficial dehiscence | 1.169 ± 0.233 | 180.1 ± 83.2 | 154.8 ± 56.0 | 28.62 ± 2.86 |
| 5 Need for negative pressure wound therapy, delayed suture removal, additional antibiotics, or surgical debridement | 1.189 ± 0.190 | 187.6 ± 76.4 | 159.2 ± 53.9 | 29.73 ± 3.81 |


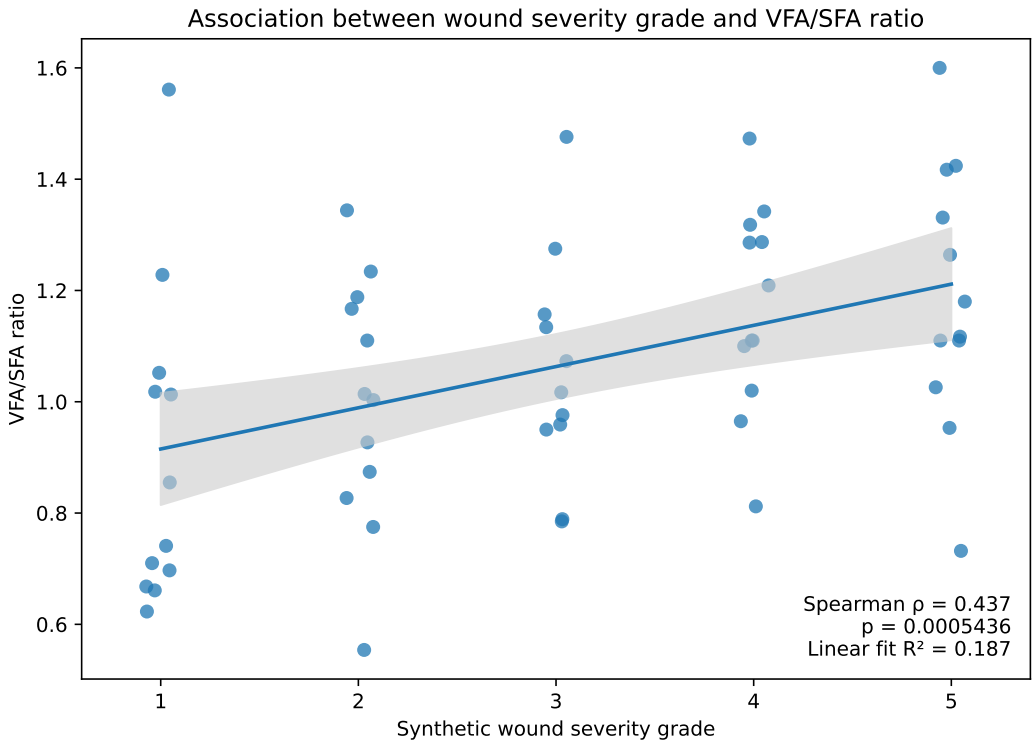


**Supplementary Figure 1. Association between wound severity grade and VFA/SFA ratio.**
Scatterplot showing the relationship between synthetic wound severity grade (1–5) and VFA/SFA ratio. The solid line represents the linear regression fit and the shaded area indicates the 95% confidence interval. Spearman analysis demonstrated a moderate positive correlation (ρ = 0.437, p = 0.00054). The linear fit yielded an R² of 0.187.
